# Hepatitis C virus NS4B protein induces epithelial-mesenchymal transition by upregulation of Snail

**DOI:** 10.1186/s12985-017-0737-1

**Published:** 2017-04-21

**Authors:** Bicheng Hu, Shenggao Xie, Yuqian Hu, Wen Chen, Xiaofan Chen, Yi Zheng, Xinxing Wu

**Affiliations:** 1 Institute of Virology, School of Basic Medical Sciences, State Key Laboratory of Virology, Wuhan University, Wuhan, 430071 Hubei China; 2 School of Laboratory Medicine, Hubei University of Chinese Medicine, Wuhan, 430065 Hubei China; 3 The Central Laboratory, Guangming New District People’s Hospital, Shenzhen, 518106 Guangdong China

**Keywords:** HCV, Epithelial to mesenchymal (EMT), Cadherin, Snail, Hippo pathway

## Abstract

**Background:**

Chronic hepatitis C virus (HCV) infection is an important cause of hepatocellular carcinoma (HCC). Epithelial to mesenchymal transition (EMT) is a key process associated with tumor metastasis and poor prognosis. HCV infection, HCV core and NS5A protein could induce EMT process, but the role of NS4B on EMT remains poorly understood.

**Methods:**

We overexpressed HCV NS4B protein in HepG2 cells or Huh7.5.1 cells infected by HCVcc, the E-cadherin expression, N-cadherin expression and the EMT-associated transcriptional factor Snail were determined. The migration and invasion capabilities of the transfected cells were evaluated using wound-healing assay. Additionally, we used Snail siRNA interference to confirm the relation of HCV NS4B and Snail on EMT promotion.

**Results:**

HCV NS4B increased the expression of EMT related markers and promoted cell migration and invasion. Snail knock-down almost completely eliminated the function of NS4B protein in EMT changes and reversed cell migration capacity to lower level. HCV NS4B protein could reduce the expression of Scribble and Hippo signal pathway were subsequently inactivated, resulting in the activation of PI3K/AKT pathway, which may be the reason for the up-regulation of Snail.

**Conclusions:**

This study demonstrates that HCV NS4B protein induces EMT progression via the upregulation of Snail in HCC, which may be a novel underlying mechanism for HCV-associated HCC development, invasion and metastasis.

## Background

The epithelial-mesenchymal transition (EMT) is a fundamental biological process by which epithelial cells lose their cell polarity and cell-cell adhesion, undergo a transition from an epithelial phenotype to a mesenchymal phenotype and gain migratory and invasive properties. It can take place in a variety of biological processes, including embryonic development, morphogenesis, tissue repair, carcinogenesis and metastasis [1, 2]. Epithelial cells can adhere to each other by all kinds of cell-to-cell junctions, such as adherens junctions, desmosomes and tight junctions. During EMT procession, cells acquire invasive and metastatic properties and easily invade through the extracellular matrix [3–5]. The underlying mechanism of EMT is associated with some transcription factors, such as Snail, TWIST and ZEB (zinc finger E-box-binding protein) families, and reduced expression of E-cadherin [6]. Zinc-finger protein Snail is a transcriptional factor of EMT that negatively regulates the expression of E-cadherin through binding to the E-box motif on the promoter region of E-cadherin [7]. Snail plays an important role in the progression of hepatocellular carcinoma (HCC) caused by HCV infection, associating with the disruption of adherens junction, invasion, metastasis and poor prognosis. Yang and coworkers reported that the increasing of snail was more frequently observed in HCC related to HCV infection than other chronic liver diseases [8]. EMT is also linked to some proteins which participate in regulation of cytoskeleton and motility, including vimentin, fibroblast-specific protein1 (FSP1), osteopontin, α-smooth muscle actin (α-SMA). Accumulating evidence suggests that hepatocellular EMT plays a pivotal role in the dissemination of malignant hepatocytes during HCC progression [9].

HCV is a member of the Flaviviridae family of viruses in the hepacivirus genus, HCV infection is a major cause of chronic liver diseases, cirrhosis and HCC. End-stage liver disease caused by chronic HCV infection is the leading cause of liver transplantation in developed countries [10]. Nearly over 170 million people are suffering from HCV infection worldwide and 3–4 million people are newly infected each year. There is no effective vaccines to prevent HCV infection, HCV can establish a long-standing persistent infection in hepatocytes [11]. The recurrence rate of HCV related HCC are much higher than other types of hepatitis virus after hepatic resection, suggesting that HCV plays a key role in carcinogenesis and metastasis [12]. HCV core protein can repress the expression of E-cadherin and up-regulate the mesenchymal phenotypic markers, such as fibronectin, vimentin and N-cadherin, driving EMT procession [13]. Moreover, recent evidence indicates HCV core protein can directly inhibit the expression of E-cadherin via hypermethylation of the promoter region of E-cadherin gene by elevating levels of DNA methyltransferase 1 and 3b [14].

HCV NS4B is a 27KD non-structural protein, plays a crucial role in HCV life cycle [15]. It is a hydrophobic, highly conserved protein. NS4B expression causes alteration of endoplasmic reticulum (ER) membrane and formation of membranous web, which can provide a platform for HCV viral replication [16]. HCV NS4B also contributes to carcinogenesis, which activates cancer-related STAT3 pathway and NF-κB transcription factor via the endoplasmic reticulum overload response [17, 18]. Our previous work had shown that HCV NS4B could interact with Scribble to facilitate its degradation via proteasome pathway [19]. Scribble, classified as a LAP protein containing four PDZ domains in its C-terminal, is a consisting member of epithelial cell polarity complex. The formation of epithelial cell polarity consists of three function complex, including the Scribble, Par and Crumbs complex [20, 21]. The Scribble polarity module is composed of three proteins, Scribble, Discs Large (Dlg) and Lethal giant larvae (Lg1), localizing at basolateral membrane domain. The Par complex localizing in tight junctions of epithelia, cooperates with Crumbs complex for establishment and maintenance of apical cell polarity, having a mutually antagonistic relationship with Scribble complex [22]. Loss of Scribble resulting in deregulation of apical-basal cell polarity can promote migration capacity of epithelia cells [23].

Hippo signaling pathway is associated with organ size, cell proliferation and tumorigenesis [24]. In mammals, Hippo pathway is an evolutionarily conserved protein kinase cascade to regulate transcription coactivator YAP (Yes-associated protein) and its homologs TAZ (transcriptional coactivator with PDZ binding motif). Once Hippo pathway was activated, MST1/2 (mammalian Ste2-like kinases) phosphorylated LATS1/2 (large tumor suppressor kinase 1/2), resulting in LATS1/2-mediated phosphorylation of YAP. Phosphorylated YAP was binding to 14-3-3 protein and was degraded by an ubiquitin-proteasome-dependent manner in cytoplasm. Hippo pathway is a vital mechanism related to the carcinogenesis and development of liver cancer. Impaired Hippo pathway signaling can lead to YAP activation and the downstream cancer-related pathway TGF-β or/and PI3K/AKT pathway are activated [25, 26]. Meanwhile, the Scribble is proved to be an upstream regulator of Hippo signaling pathway [27]. So, a hypothesis is proposed that Hippo pathway may be a bridge connecting Scribble and HCV NS4B protein related EMT procession.

In this study, we demonstrated that HCV NS4B played a critical role in EMT procession by upregulation of Snail. And our study also proved our hypothesis that Scribble-Hippo-PI3K/AKT axis was a crucial molecular mechanism in HCV NS4B caused EMT changes.

## Methods

### HCV infection system

The plasmid pFL-J6/JFH1 contained HCV J6/JFH-1 cDNA from a Japanese patient with fulminant hepatitis behind a T7 promoter, which was lineared at the 3′ end of the HCV cDNA by XbaI digestion [28]. The linear plasmid DNA was used as a template to create HCV RNA. HCV RNA was transfected into Huh7.5.1 cells. Virus could be harvested from cell culture supernatants, which was filtered through a 0.45 μm pore size membrane. HCV RNA level of the supernatants was quantified by real-time PCR (ABI 7300). The viral titer was determined by the average number of HCV NS5A positive foci at the highest dilutions as described previously [29].

### The construction of plasmids

The pEGFPC1 was purchased from Invitrogen (Invitrogen, Carlsbad, CA, USA). Full length of NS4B gene with HCV genotype 1b was generated from Con1 plasmid which was kindly provided by Prof. Ralf Bartenschlager (University of Heidelberg, Germany) by using PCR. Then full length of NS4B was inserted into pEGFPC1 plasmid to construct pEGFPC1-NS4B plasmid.

### Transient transfection

The HepG2 cells were maintained in Dulbecco’s modified Eagle medium (DMEM) with 10% fetal bovine serum (FBS) and cultured at 37 °C in a humidified atmosphere with 5% CO_2_. When cells reached approximately 90% confluence in six well cell culture plate, about 1 × 10^6^ cells were transfected with 3 μg plasmids (1 and 5 μg as gradient contrast) by 5 μl lipofectamine-2000 (Invitrogen, Karlsruhe, Germany) in 150 μl DMEM medium without 10% FBS, according to manufacturers’ instructions. After 48 h, the cells were harvested and washed twice with ice-cold PBS for western blot or other experiments. Snail or control siRNA and plasmids were co-transfected into HepG2 cells at approximately 80% confluence by 5 μl lipofectamine-2000 (Invitrogen, Karlsruhe, Germany) in 150 μl DMEM medium without FBS, according to manufacturers’ instructions.

### Western blotting analysis

Transfected or HCVcc infected cells were harvested and washed twice with ice-cold PBS and then lysed in sodium dodecyl sulfate-polyacrylamide gelelectrophoresis (SDS-PAGE) loading buffer (50 mM Tris-HCl, 2% SDS, 0.1% Bromophenol blue, 10% Glycerine, 1% 2-Mercaptoethanol) for 5 min. Lysates were heated at 100 °C for 20 min, then proteins were separated on SDS-PAGE gel and were transferred into polyvinylidene fluoride (PVDF) membrane (Immobion^®^-P Transfer Membrane). The membrane was blocked with 5% nonfat milk in TBS-tween (TBS-T) solution and incubated with primary antibodies overnight at 4 °C. Then the membrane was washed with TBS-T for three times and incubated with secondary antibodies (KPL) at room temperature for 2 h. After washed three times with TBS, the protein was visualized by SuperSignal^®^ West Pico Chemiluminescent Substrate (Thermo Scientific).

### Total RNA extraction and qRT-PCR

Total RNA was extracted from cells with Trizol reagent (TaKaRa) according to the manufacturer’s instructions. Reverse transcription was conducted following the protocol of Prime Script^®^ RT Master Mix Perfect Real Time (Takara) and qPCR was performed as the instruction of SYBR® Premix Ex TaqTM II (Takara) on the ABI 7300 Real Time PCR System (Applied Biosystems). β-actin was used as the internal control. The Primers were designed according to intron-crossing principle to remove the interference of genome DNA (Table 1). Each sample was performed in triplicate and the results represent mean ± SE.

**Table 1:** Primers of EMT related genes

Gene	Primers
E-cadherin	Sense: 5′-AACAGGATGGCTGAAGGTGACA-3′
	Antisense: 5′- GTAAGCGATGGCGGCATTGTAG-3
N-cadherin	Sense: 5′- AGAACGCCAGGCCAAACAAC-3′
	Antisense: 5′- ATTCGTCGGATTCCCACAGG-3′
Snail	Sense: 5′- TGAGGCCAAGGATCTCCAGG-3′
	Antisense: 5′-AAGGGCTTCTCGCCAGTGTG-3′
β-actin	Sense: 5′-TACGCCAACACAGTGCTGTCTG-3′
	Antisense: 5′-CACATCTGCTGGAAGGTGGACA-3′

### Immunofluorescence analysis

At 24 h post-transfection, cells were washed twice with ice-cold PBS and fixed in 4% paraformaldehyde for 15 min at room temperature. Cells were permeabilized in 0.1% Triton X-100 in PBS for 3–5 min, blocked with 3% FBS in PBS for 1 h, and then incubated with primary antibody overnight at 4 °C followed by incubated with secondary antibody conjugated to red fluorochromes for 1 h at room temperature. The cell nuclei were stained with 1 μg/ml dihydrochloride (Beyotime, China) for 15 min at room temperature. Cover slips were mounted upside down on slides with suitable volume of ProLong Gold Antifade Reagent (Invitrogen, USA). After that, the cells were examined using an OLYMPUS IX81 fluorescence microscope equipped with the appropriate filter sets and OLYMPUS FluoView Ver.2.0a Viewer software.

### Wound-healing assay

Cells grown to 90% confluence in 6-well plates were mechanically injured with a sterile micropipette tip. The photographs were taken at 0, 12, and 24 h and wound healing rate was evaluated by measuring wound closure rate.

### Statistical analysis

Experimental data were analyzed using GraphPad Prism 4 (GraphPad Software, La Jolla, CA, USA). The value was presented as means ± S.D. Two-tailed student’s t-test was used for all statistical analysis; a *P* value of less than 0.05 was considered as statistically significance.

## Results

### HCVcc-infected cells presented the changes of EMT and upregulation of Snail

**Fig. 1:**
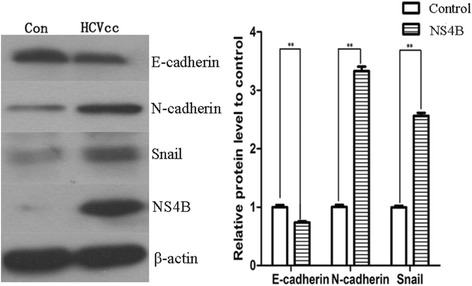
HCVcc-infected cells presented the changes of EMT. The expression of E-cadherin, N-cadherin and Snail in Huh7.5.1 cells (Control group) and HCVcc infected Huh7.5.1 cells (HCVcc group). *P < 0.05, **P < 0.01

Huh7.5.1 cells were infected with HCVcc for 48 h and the cells not infected as control group. Then cells were harvested for western blot assay. In HCVcc infected cells, the expression of epithelial phenotypic markers E-cadherin was decreased whereas mesenchymal phenotypic marker N-cadherin was increased compared with the control group (Fig. 1). Meanwhile, we also observed the upregulation of Snail, one of the most important transcription factors associated with EMT. These results revealed that EMT process really occurred in HCVcc infection cells and the initiation of EMT may be due to upregulation of Snail.

### HCV NS4B protein induced hepatocellular EMT

**Fig. 2:**
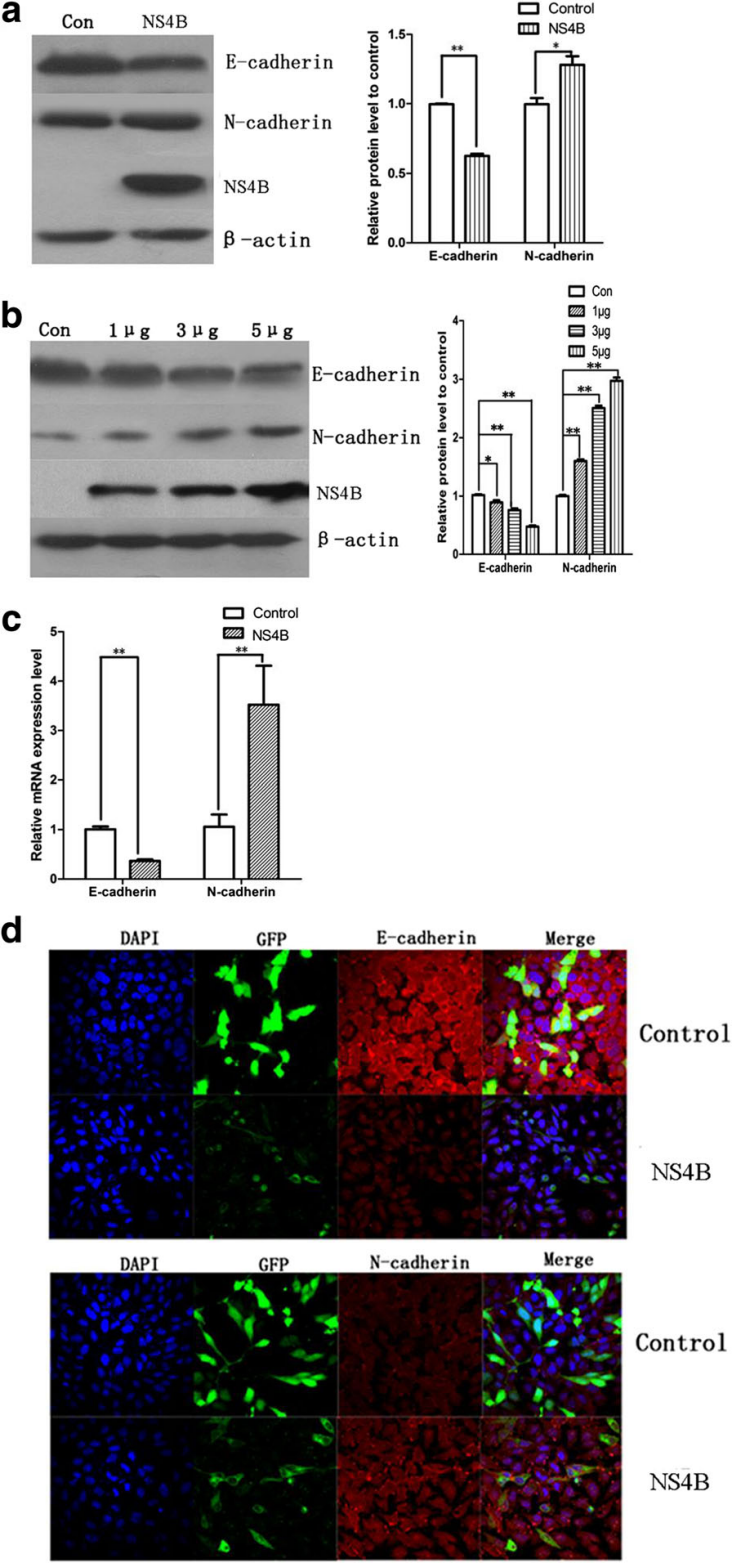
HCV NS4B induced hepatocellular EMT. **a** HepG2 cells were transfected with pEGFPC1 plasmid (control group) and pEGFPC1-NS4B plasmid (NS4B group), 48 h later, the expression of E-cadherin and N-cadherin in control group and NS4B group. **b** HepG2 cells were transfected with pEGFPC1 plasmid (control group), 1, 3, 5 μg pEGFPC1-NS4B plasmid respectively, 48 h later, the expression of E-cadherin and N-cadherin in each group. **c** qRT-PCR analysis the mRNA expression of E-cadherin and N-cadherin in control group and NS4B group. **d** Immunofluorescence analysis of E-cadherin and N-cadherin in control group and NS4B group. *P < 0.05, **P < 0.01

To investigate the effect of HCV NS4B protein on hepatocellular EMT, we transfected empty vector or pNS4B plasmids into HepG2 cells, and then we examined the hallmarks of EMT at 48 h. It was found that cells transfected with pNS4B plasmids had lower expression of E-cadherin and higher expression of N-cadherin than the cells transfected with empty vector, as similar to the EMT phenotype in HCVcc infected cells, suggesting that HepG2 cells were in EMT procession (Fig. 2a). Furthermore, we transfected empty vector, pNS4B plasmids (1, 3, 5 μg) in HepG2 cells to investigate whether the EMT process was related to NS4B levels, at 48 h post transfection, we found that the expression of E-cadherin decreased gradually and N-cadherin upregulated, along with the levels of pNS4B plasmids increased. These results indicated that HCV NS4B was the reason for HepG2 cell EMT (Fig. 2b). In addition, the qRT-PCR results showed that E-cadherin mRNA levels were decreased and the expression levels of N-cadherin increased after pNS4B plasmids transfection (Fig. 2c). Immunofluorescence analysis also confirmed the decreased expression of epithelial marker E-cadherin and increased expression of mesenchymal marker N-cadherin (Fig. 2d). These results demonstrated that NS4B contributed to promoting HepG2 cells EMT.

### Upregulation of Snail was coupled with the process of HCV NS4B promotion-EMT

**Fig. 3:**
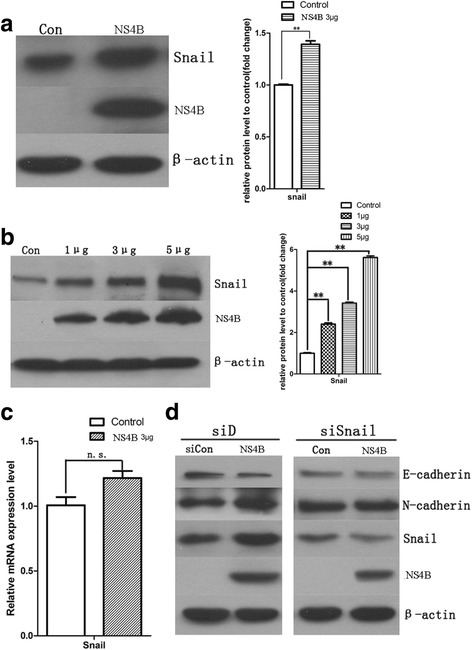
HCV NS4B promoted EMT due to upregulation of Snail. **a** HepG2 cells were transfected with pEGFPC1 plasmid (control group) and pEGFPC1-NS4B plasmid (NS4B group), 48 h later, the expression of Snail in control group and NS4B group. **b** HepG2 cells were transfected with pEGFPC1 plasmid (control group), 1, 3, 5 μg pEGFPC1-NS4B plasmid respectively, 48 h later, the expression of Snail in each group. **c** qRT-PCR analysis the mRNA expression of Snail in control group and NS4B group, The HepG2 cells were transfected with 3 μg pEGFPC1-NS4B plasmids in the qRT-PCR assay. **d** Western blotting analysis the expression of E-cadherin, N-cadherin, snail and HCV-NS4B in plasmids transfected cells, Snail siRNA for knockdown Snail. Co-transfected Snail siRNA and 3 μg pEGFPC1-NS4B plasmids into HepG2 cells, the expression level of Snail in HepG2 cells decreased after Snail siRNA transfection, and the phenomenons that downregulation of E-cadherin and upregulation of N-cadherin reversed while knockdown of snail. Con ~ for control group, n.s. ~ for no significant different, *P < 0.05, **P < 0.01

It was identified that upregulation of Snail could induce EMT changes [30]. We transfected empty vector, pNS4B plasmids (1, 3 and 5 μg) into HepG2 cells, we observed the expression of Snail was increased compared with the empty vector in the HepG2 cells after HCV NS4B-transfected for 48 h (Fig. 3a), meanwhile, we also observed a positive correlation between the upregulation of Snail levels and pNS4B plasmids levels (Fig. 3b). But the levels of Snail mRNA that we measured after pNS4B plasmids transfected for 48 h had no significant difference with the empty vector (Fig. 3c), suggesting the upregulation of Snail may rely on a translation-dependent manner. In order to confirm the effect of snail on EMT process, we co-transfected Snail siRNA and NS4B into HepG2 cells, the expression level of Snail in HepG2 cells decreased after Snail siRNA transfection, and we found the phenomenons that downregulation of E-cadherin and upregulation of N-cadherin reversed while knockdown of snail. These results provided a powerful evidence that snail played a vital role in NS4B-induced EMT changes (Fig. 3d).

### HCV NS4B protein promoted hepatoma cell migration and invasion

**Fig. 4:**
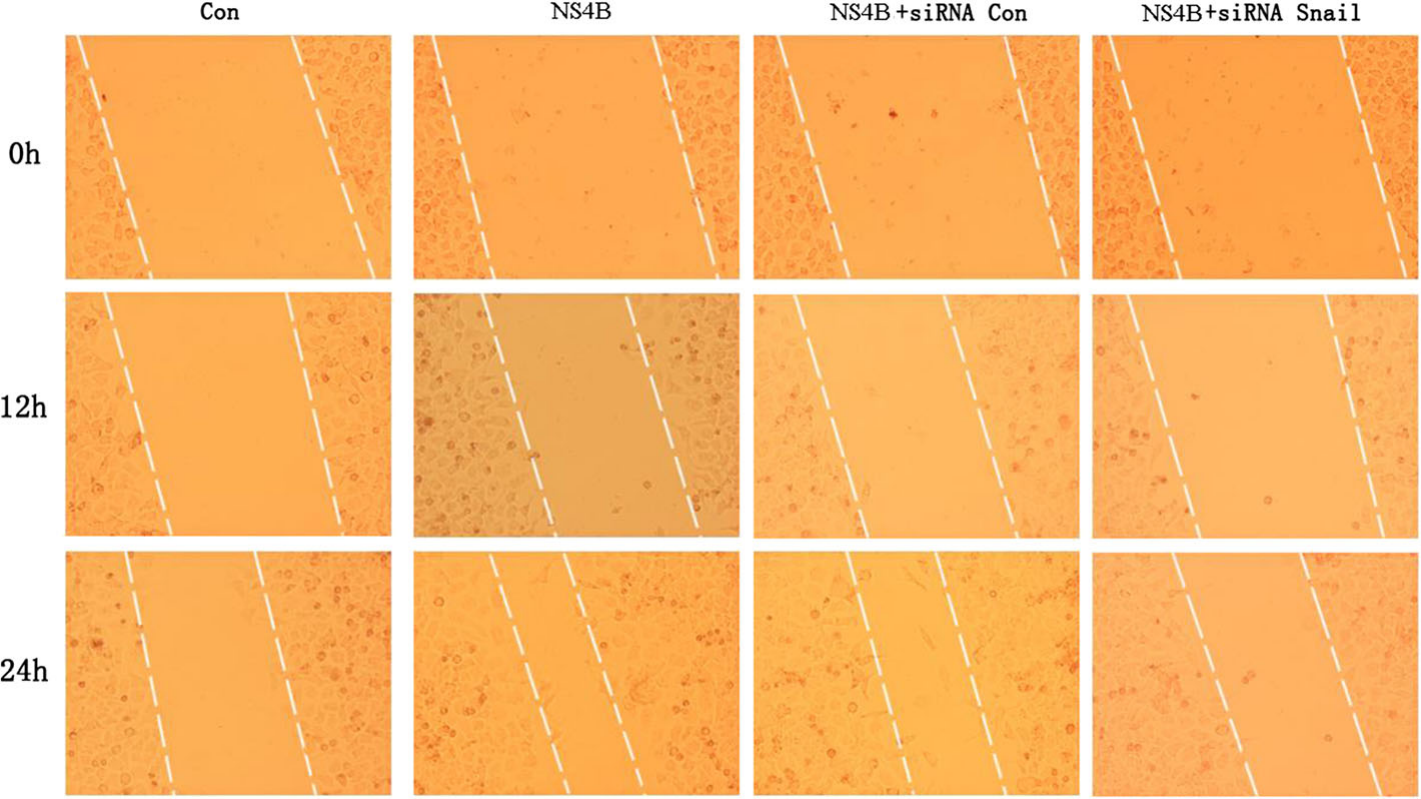
HCV NS4B promoted hepatoma cell migration and invasion. Would healing assay were used to examine the migration and invasion capabilities of the HepG2 cells were transfected with HCV NS4B, cotransfected snail or control siRNA and NS4B. Microscopic observation of cell migration and recorded at 0, 12 and 24 h after scratching

The wound healing assay was used to examine the migration and invasion capabilities of the HepG2 cells transfected with HCV NS4B. In the same period of time, the HepG2 cells with HCV NS4B transfected expansion to fill the gap further, indicated that HCV NS4B promoted HepG2 cell motility and invasiveness. To test whether HCV NS4B transfected cells acquired migration and invasion capabilities through upregulation of Snail, we cotransfected snail or control siRNA and NS4B plasmid into HepG2 cells. We found that the increased migration in NS4B transfected cells was reversed by snail siRNA treatment, cells cotransfected with Snail siRNA showed slower healing of the wound in comparison to the cells treated with control siRNA, which meant that snail played an important role in HCV NS4B induced hepatoma cell migration and invasion (Fig. 4).

### Upregulation of Snail may be caused by Scribble-Hippo-PI3K/AKT pathway

**Fig. 5:**
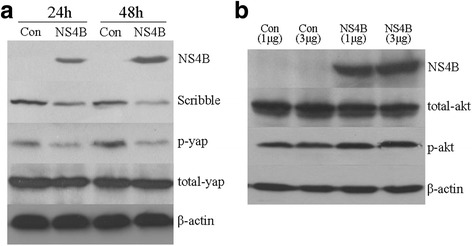
Western blotting analysis the key elements of Scribbe-Hippo-PI3K/AKT pathway in the HepG2 cells with HCV NS4B transfected. **a** HepG2 cells were transfected with pEGFPC1 plasmid (control group) and pEGFPC1-NS4B plasmid (NS4B group), 24 and 48 h later, the expression of Scribble, p-Yap and total Yap in control group and NS4B group. **b** HepG2 cells were transfected with 1, 3 μg pEGFPC1 plasmid and 1, 3 μg pEGFPC1-NS4B plasmid, 48 h later, the expression of p-akt, total-akt in each group

The PI3K/AKT pathway can enhance the stability of Snail via phosphorylation at its two serine residues, and then upregulate its activity by post-translational modification [31]. Therefore, we investigated whether upregulation of Snail by HCV NS4B expression was related to Scribble-Hippo-PI3K/AKT pathway. The key elements in this pathway including Scribble, p-Yap, total-Yap, p-Akt and total-Akt were examined by Western blot. In the HepG2 cells tranfected by HCV NS4B for 24 and 48 h, the expression of Scribble and p-Yap was decreased, but the total Yap had no significant difference. So the ratio of P-Yap/total Yap was decreased, these results suggested NS4B expression in HepG2 cells inhibited the activity of Hippo signal pathway, additionally the down-regulation of Scribble and p-Yap were in a time dependent manner (Fig. 5a). Then we further used HepG2 cells transfected with pNS4B plasmids (1, 3 μg) for 48 h to evaluated the expression of p-Akt and total-Akt, the results showed that p-Akt was increased and total-Akt was decreased. Taken together, these results demonstrated the activation of PI3K/AKT pathway in HepG2 cells with NS4B expression (Fig. 5b).

## Discussion

EMT is a key event in carcinogenesis and EMT has been confirmed to play an important role during HCC progression, whereby epithelial cells lose apical-basal polarity and cell junctions and acquire a mesenchymal phenotype with invasion and metastasis properties. EMT of malignant hepatocytes correlate well with malignancy and poor prognosis [32, 33]. HCV infection was identified to induce EMT in primary hepatocytes and cell lines [13]. Many studies had proved that HCV core or NS5A protein could induce EMT process in hepatoma cells by different kinds of manner [34–36]. Our study firstly observed that NS4B was also involved in EMT of hepatocytes, the underlying molecular mechanism remains elusive and needs to be fully understood in HCC progression.

E-cadherin is a phenotypic membranous adhesion molecule on epithelial cells. It has been proved to be involved in proliferation, migration and the process of EMT, keeping cells in a quiescent, non-motile state. N-cadherin is a molecular marker of acquisition of a motile mesenchymal phenotype and involves in the EMT and metastasis of cancer cells. The loss of E-cadherin and gain of N-cadherin expression is considered as the most common hallmark of EMT. It was reported that the expression of E-cadherin was reduced significant in HCV-caused HCC compared to normal tissues [37]. In the present study, HCV infection or ectopic expression of HCV NS4B in HepG2 cells reduced E-cadherin expression and increased N-cadherin expression. Furthermore, HCV NS4B expression contributed to migration and invasion abilities in hepatoma cells. This study showed high levels of Snail expression in HCV NS4B-transfected HepG2 cells and HCVcc-infected Huh7.5.1 cells. When the snail knockdown by siRNA, the increased migration in NS4B transfected cells was reversed accordingly, moreover, the degree of downregulation of E-cadherin and upregulation of N-cadherin attenuated, which meant that Snail played an important role in HCV NS4B induced EMT.

The precise molecular mechanism of the Snail regulation is still unclear, previously, our teams have proved that HCV NS4B targets Scribble for proteasome-mediated degradation to facilitate cell transformation [19]. Scribble is an epithelial polarity protein plays a critical role in establishing and maintaining epithelial cell adhesion, polarity and proliferation, which serves as a tumor suppressor [38]. Loss of Scribble can lead to EMT. Yamben and coworkers reported that deletion Scrib (a mouse homolog of Scribble) in the lens resulted in downregulation of E-cadherin, apical polarity protein ZO-1 (Zonula occludens-1) and upregulation of Snail [39]. In this study, Scribble was decreased in HCV NS4B transfected HepG2 cells and impaired Hippo pathway signaling, resulting in the downregulation of the ratio of p-Yap/total-Yap. Hippo pathway played an important role in the development of HCC. High-level expression of Yes-associated protein (YAP) was more frequently observed in malignant liver tissues than normal liver tissues [40, 41]. Scribble acted as an upstream regulator of Hippo signal pathway and caused deregulation of the Hippo tumor suppressor pathway. Verghese and colleagues reported that Scribble interacted with Expanded and Dachs to regulate Warts level and stability in Drosophila, and then placing Scribble in Hippo pathway network [27]. The nuclear effector of the Hippo Pathway YAP and transcription enhancer factor TEAD acted as a conserved enhancer directly targeting the first intron of PIK3CB (Phosphoinositide 3-Kinase Catalytic Beta Polypeptide), encoding the catalytic subunit p110β of human phosphatidylinositol 3-kinase (PI3K) to activate the PI3K/AKT pathway [26]. In present study, we demonstrated that HCV NS4B suppressed Hippo pathway and activated the PI3K/AKT pathway. Hippo pathway acted as a bridge between the downregulation of Scribble and activation of PI3K/AKT pathway. It had already been proved that PI3K/AKT pathway regulated the stability of Snail to promote EMT. These results suggested that Scribble-Hippo-PI3K/AKT pathway maybe involved in upregulation of Snail.

## Conclusions

**Fig. 6:**
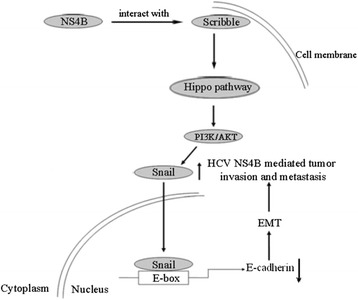
The delineation of a potential molecular mechanism underlying EMT caused by HCV NS4B

In summary, our study identified that overexpression of HCV NS4B in HepG2 cells could lead to EMT-like changes, which enhanced migration and invasion of hepatoma cells. The mechanism underlying EMT caused by HCV NS4B were due to upregulation of Snail. Snail knockdown could reverse the procession of EMT and inhibited the migration of HepG2 cells. The regulation of Snail would probably be mediated through Scribble-Hippo-PI3k/AKT pathway (Fig. 6). Our work revealed a potential molecular mechanism underlying EMT caused by HCV NS4B. It may provide novel insight into potential new therapeutic approaches for patients with HCV infection.

## Abbreviations

AKT: Protein kinase B
Dlg: Discs Large
DMEM: Dulbecco’s modified Eagle medium
EMT: Epithelial to mesenchymal transition
ER: Endoplasmic reticulum
FBS: Fetal bovine serum
FSP: Fibroblast-specific protein
HCC: Hepatocellular carcinoma
HCV: Hepatitis C virus
LAP: Leucine-rich repeats and PDZ domains
LATS1/2: Large tumor suppressor kinase 1/2
Lg1: Lethal giant larvae
MST1/2: Mammalian Ste2-like kinases
NF-κB: Nuclear factor kappa-light-chain-enhancer of activated B cells
NS4B: Non-structural protein 4B
PDZ: PSD-95/Dlg-A/ZO-1
PI3K: Phosphoinositide 3-Kinase
PIK3CB: Phosphoinositide 3-Kinase Catalytic Beta Polypeptide
PVDF: Polyvinylidene fluoride
SDS-PAGE: Sodium dodecyl sulfate-polyacrylamide gelelectrophoresis
SMA: Smooth muscle actin
STAT: Signal transducer and activator of transcription
TAZ: Transcriptional coactivator with PDZ binding motif
TEAD: Transcriptional enhancer factor domain family member
TGF-β: Transforming growth factor-β
YAP: Yes-associated protein
ZEB: Zinc finger E-box-binding protein
ZO-1: Zonula occludens-1
